# Sprouty is a cytoplasmic target of adenoviral E1A oncoproteins to regulate the receptor tyrosine kinase signalling pathway

**DOI:** 10.1186/1743-422X-8-192

**Published:** 2011-04-26

**Authors:** Angelika Zaremba, Ursula Schmuecker, Helmut Esche

**Affiliations:** 1 Laboratory of Signal Transduction, National Institute of Environmental Health Sciences, Research Triangle Park, PO Box 12233, Durham, NC 27709, USA; 2 Institute for Molecular Biology (Cancer Research), University of Essen, Medical School, Hufelandstrasse 55, 45122 Essen; Germany

## Abstract

**Background:**

Oncoproteins encoded by the early region of adenoviruses have been shown to be powerful tools to study gene regulatory mechanisms, which affect major cellular events such as proliferation, differentiation, apoptosis and oncogenic transformation. They are possesing a key role to favor viral replication via their interaction with multiple cellular proteins. In a yeast two-hybrid screen we have identified Sprouty1 (Spry1) as a target of adenoviral E1A Oncoproteins. Spry proteins are central and complex regulators of the receptor tyrosine kinase (RTK) signalling pathway. The deregulation of Spry family members is often associated with alterations of the RTK signalling and its downstream effectors, leading to the ERK pathway.

**Results:**

Here, we confirm our yeast two-hybrid data, showing the interaction between Spry1 and E1A in GST pull-down and immunoprecipitation assays. We also demonstrated the interaction of E1A with two further Spry isoforms. Using deletion mutants we identified the N-terminus and the CR conserved region (CR) 3 of E1A- and the C-terminal half of Spry1, which contains the highly conserved Spry domain, as the essential sites for direct interaction between Spry and E1A. Immunofluorescent microscopy data revealed a co-localization of E1A_13S _with Spry1 in the cytoplasm. SRE and TRE reporter assays demonstrated that co-expression of Spry1 with E1A_13S _abolishes the inhibitory function of Spry1 in RTK signalling, which is consequently accompanied with a decrease of E1A_13S_-induced gene expression.

**Conclusions:**

These results establish Spry1 as a cytoplasmic localized cellular target for E1A oncoproteins to regulate the RTK signalling pathway, and consequently cellular events downstream of RTK that are essential for viral replication and transformation.

## Background

Proteins encoded by the early transcription unit 1A (E1A) of Adenovirus (Ad) are essential for the viral life cycle because of their necessity in regulating the expression of all other viral genes [1]. In addition these proteins modulate the expression of specific cellular genes in infected cells to facilitate viral reproduction [2,3]. E1A oncoproteins cooperate with Ad early region 1B (E1B) oncogene products to transform rodent cells in culture and, depending on the serotype, to induce tumors in immunocompetent animals (e.g. Ad12) [4-6]. Ad12 E1A gives rise to five proteins of which the 266R protein (translated from a 13S mRNA; henceforth referred to as E1A_13S_) and the 235R protein (translated from a 12S mRNA; henceforth referred to E1A_12S_) are the predominant isoforms [2,7]. Both proteins are translated in the same reading frame but differ in a short stretch of 31 aa, called CR3, that is absent in E1A_12S_. CR3 is one of four E1A regions (CR1, CR2, CR3 and CR4) that are highly conserved among all Adenovirus serotypes [2]. The N terminus and the CRs of E1A mediate most of the gene regulatory functions necessary for viral reproduction and transformation [8]. Due to the lack of a sequence-specific DNA binding activity, E1A proteins, mainly known as transcription factors, fulfill their gene regulatory functions by interaction with cellular transcription factors such as c-Jun, ATF, CREB, or repressors such as pRB and cellular co-factors like p300 and CBP [9-12].

The idea that E1A is also capable of exerting its regulatory functions by directly affecting cytoplasmic processes was supported by the discovery that a certain amount of E1A proteins is acetylated at Lys^239^, which determines the cytoplasmic localization of E1A proteins by interfering with the nuclear transport [13]. Until now, a few cytoplasmic localized interaction partners of E1A have been identified including the regulatory subunit II of protein kinase A (PKA-RIIα) [14], the receptor for activated C-kinase l (Rackl) [15,16], and the cytoplasmic proteasome 26S [17].

Sprouty (Spry) proteins have been identified as regulatory proteins of the receptor tyrosine kinase (RTK) signalling pathway [18-21]. They appear to play an inhibitory role in many cellular events due to their effect on RTK, especially in FGF-dependent developmental processes [22-25]. First identified in *Drosophila *[19], Sprouty homologues have been discovered in human and mouse. A high degree of conservation of key functional amino acids has been shown for Spry proteins among different species [20,22,26,27].

A unique and highly conserved C-terminal cysteine-rich Spry domain has been identified for all 4 mammalian Spry isoforms. The Spry domain is responsible for palmitoylation at the plasma membrane. Mutations in this region disrupt membrane localization and abrogate Spry functions [22,28,29]. A conserved short N-terminal tyrosine-containing motif of Spry was discovered to be critical for physiological functions to inhibit FGF signalling and sustain EGF signalling [30,31]. Different interacting partners have been identified, which upon binding with Spry consequently influence RTK signalling pathway, including Rafl, Grb2, c-Cbl and Shp2 [28,31-33]. The deregulation of Sprouty was described in a number of cancers [34-38].

In a search to identify potential cytoplasmic binding partners of Adenovirus E1A oncoproteins we detected Spry1 as a putative binding partner of E1A. We were able to confirm this interaction in GST pull-down and immunoprecipitation assays. We also demonstrated an interaction of two further Spry isoforms with E1A and characterized the protein domains that are responsible for binding. Using confocal immunofluorescence microscopy, we detected a co-localization of Spry1 and E1A_13S _exclusively in the cytoplasm. Co-expression of E1A_13S _with Spry1 indicated a functional role for this interaction to modulate RTK signalling pathway and thereby to regulate cellular processes.

## Results

### Sprouty proteins interact with E1A isoforms

**Figure 1:**
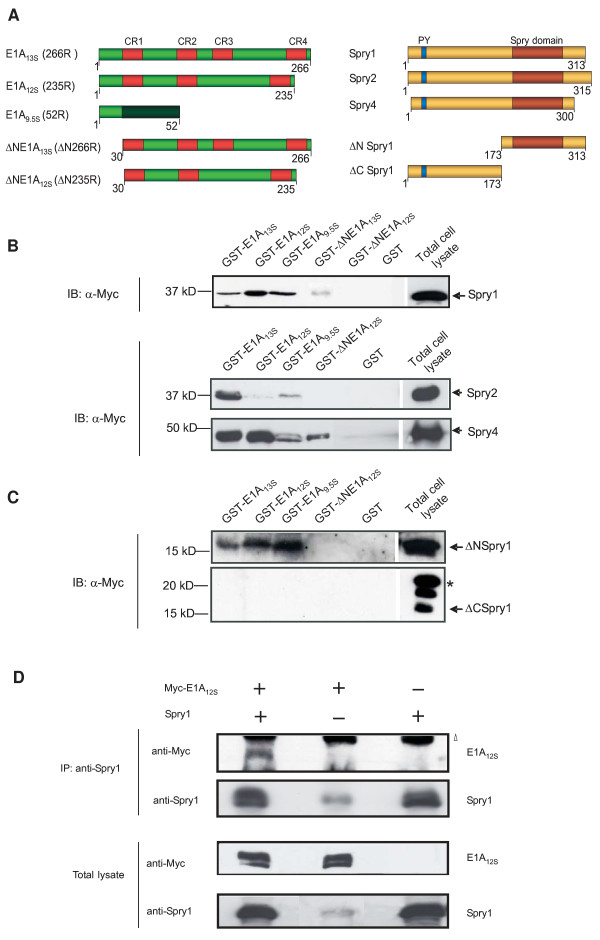
**Sprouty proteins interact with E1A isoforms**. (A) Schematic representation of the E1A wild type isoforms E1A_13S_, E1A_12S_, E1A_9.5S_, the deletion mutants ΔNE1A_13S _and ΔNE1A_12S _(with a deletion of the first 29 N-terminal amino acids) and Spry isoforms and deletion mutants (ΔNSpry1 with a deletion of amino acids 1-173; ΔCSpry1 with a deletion of amino acids 174-313). Red boxes in E1A proteins represent the conserved regions 1 to 4; the brown box in Spry represents the Spry domain and the blue box the conserved tyrosine phosphorylation site. (B, C) For GST pull-down assays HeLa cell extract, containing the isoform Spry1, Spry2 or Spry4 (B) or the Spry-mutants ΔNSpry1 or ΔCSpry1 (C) were incubated with the protein leader sequence GST- or GST-E1A-fusion proteins as indicated. 0.5 mg of cellular lysate and 40 μg of GST-E1A or GST were incubated at 4°C for 1 h. Proteins bound were subjected to SDS-PAGE, and immunoblot analysis was performed with antibodies directed against the Myc-epitope of Spry-fusion proteins. * The higher molecular weight band may represent post-translational modification of Spry1 (palmitoylation and phosphorylation) as reported before [22]. (D) For immunoprecipitation HeLa cells were transiently co-transfected with Spry1 and Myc-tagged E1A_12S _expression vectors as indicated. Cell extracts were prepared 48 h post transfection and incubated with anti-Spry1 antibodies. Interacting proteins were precipitated and analyzed on SDS-PAGE by Western blotting using anti-Spry1 and anti-Myc antibodies. Transient expression was examined in the total lysate (Δ co-eluted antibody heavy chains).

In a previous search for cellular targets of adenoviral proteins using a mouse embryo cDNA-expression library (Chevrayx and Nathans, Howard Huges Medical Institute, Baltimore) and a SOS-yeast two hybrid system we detected mouse Spry1 as a cytoplasmic interaction partner of Ad12 E1A proteins (data not shown). In order to confirm this observation, we first examined the binding of Spry1 and further Spry proteins with Ad12 E1A isoforms by GST pull-down assays. Mouse Spry1, Spry2 and Spry4 were exogenously expressed in HeLa cells and incubated with E1A_13S_,-, E1A_l2S_- and E1A_9,5S_-GST-fusion proteins (Figure 1A). Spry1 and Spry4 interact with all three Ad12 E1A isoforms (Figure 1B). For Spry2 we were not able to detect an interaction with E1A_12S _and E1A_9.5S _showed only weak binding. The mutant ΔNE1A_12S_, an isoform where we deleted the first 29 amino acids of the N terminus, showed no interaction with Spry1 and Spry2 (Figure 1B). However, for Spry4 we were able to detect an interaction with ΔNE1A_12S_, indicating an essential function of the Ad12 E1A N-terminal domain for efficient interaction with Spry1 and Spry2. Although weak, we surprisingly detected an interaction between Spry1 and the analog aminoterminal deletion mutant of the E1A_13S _isoform, indicating that the conserved region 3 (CR3) is also involved in binding with Spry1. However the domain of E1A that is responsible for the interaction with Spry4 remains to be elucidated. To further define the region of Spry1 that is necessary for binding with E1A we constructed two deletion mutants in which we truncated either the N-terminal (ΔNSpry1) or the C-terminal half of Spry1 (ΔCSpry1) (Figure 1A). Our data showed that the C-terminal half of Spry1, bearing the highly conserved Spry domain, is necessary for the interaction with E1A, whereas the tyrosine-containing sequence of the N-terminal part is not essential for interaction (Figure 1C). Since the Spred family of proteins are likewise Sprouty-domain-containing proteins that show regulatory functions in RTK signalling pathway [39,40], we studied the interaction of E1A proteins with mouse Spred1 and Spred2 by GST pull-down assays. In our experiments, we were not able to detect any interactions between Spred proteins and E1A isoforms (data not shown). It's possible that conformational changes due to additional binding domains [such Ena/Vasp homolog (EVH)1 and c-Kit binding (KBD)-domain] in Spred proteins prevent E1A from binding with this protein family [20].

To confirm whether the interaction of E1A with Spry occurs in cells we co-expressed Spry1 and Myc-tagged E1A_12S _proteins in HeLa cells. Cell lysates were subjected to immunoprecipitation with anti-Spry1 antibody, and the immunoprecipitates were then analyzed for the presence of Myc-tagged E1A_12S. _Results from these experiments have confirmed that E1A_12S _binds Spry1 in mammalian cells (Figure 1D).

**Figure 2:**
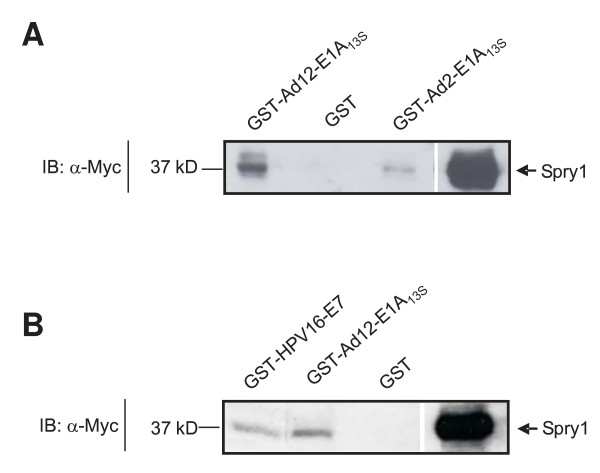
**Spry1 interacts with E1A_13S _of the non-oncogenic Adenovirus type 2 (Ad2) and with the Human papillomavirus type 16 (HPV16) E7 protein**. Lysates from cell extracts expressing Spry1 were incubated with (A) GST-Ad2-E1A_13S _and (B) GST-HPV16-E7-fusion proteins or for control reactions with the leader sequence GST or GST-Ad12-E1A_13S _as indicated. The assays were performed with 0.5 mg of cellular lysate and 40 μg of GST-fusion proteins at 4°C for 1 h. The bound proteins were subjected to SDS-PAGE and analyzed by immunoblotting. Spry1 was detected by an antibody directed against the Myc-epitope of the Spry-fusion protein.

To verify if the interaction with Spry proteins is restricted to E1A proteins of the highly ongogenic adenovirus serotype 12 (Ad12), we examined the interaction of the non-oncogenic adenovirus serotype 2 (Ad2) E1A_13S _protein with Spry1 and were able to detect an interaction (Figure 2A).

### Spry1 interacts with the Human papillomavirus type 16 (HPV16) E7 protein

The E7 oncoprotein of the Human Papillomavirus type 16 (HPV16-E7) displays partial amino acid sequence homology, comparable function, and similar interaction partners with adenoviral E1A oncoproteins [41,42]. Therefore, we examined and confirmed the interaction of Spry1 with the HPV16-E7 protein by GST pull-down assay (Figure 2B). As a positive control we used GST-E1A_13S _and as a negative control we used GST in the pull down assay. Our results establish Spry proteins as potential targets of presumably various DNA tumor virus oncoproteins.

### Spry1 co-localizes with E1A_13S _in the cytoplasm

**Figure 3:**
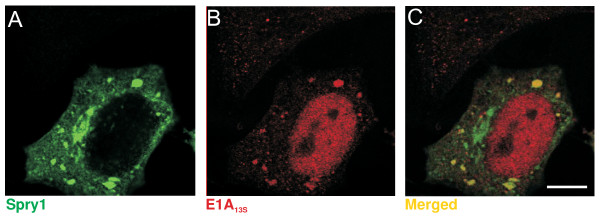
**Spry1 co-localizes with E1A_13s _in the cytoplasm**. The subcellular localization of Spry1 and E1A_13s _was determined in HeLa cells by confocal immunofluorescence microscopy using antibodies that specifically recognize Spry1 (green) and the Myc-tagged E1A_13s _(red) protein constructs. (A) Spry1 and (B) E1A_13s _transfected cells were left serum starved overnight and stimulated with 20 ng/ml bFGF for 2 h. (C) Images were overlaid for comparison of staining, yellow indicates co-localization of the two proteins. Scale bar, 10 μm.

To determine the subcellular localization of Spry1 in the presence of E1A proteins, we performed confocal immunofluorescence microscopy using antibodies that specifically recognize Spry1 and Myc-tagged E1A_13S_. HeLa cells were serum-deprived overnight and treated with bFGF for 2 h. Whereas Spry1 was distributed within the whole cytoplasm, E1A_13S _was predominantly found in the nucleus (Figure 3) with a lesser amount of E1A_13S _detectable in the cytoplasm. This cytoplasmic pool of E1A_13S _strongly co-localized with Spry1, predominantly in vesicular structures within the cytoplasm (Figure 3C). In addition, the subcellular localization of Spry1 is not affected by the presence of E1A proteins.

### Co-expression of Spry1 decreases E1A_13S_-induced gene transactivation of TRE and SRE

**Figure 4:**
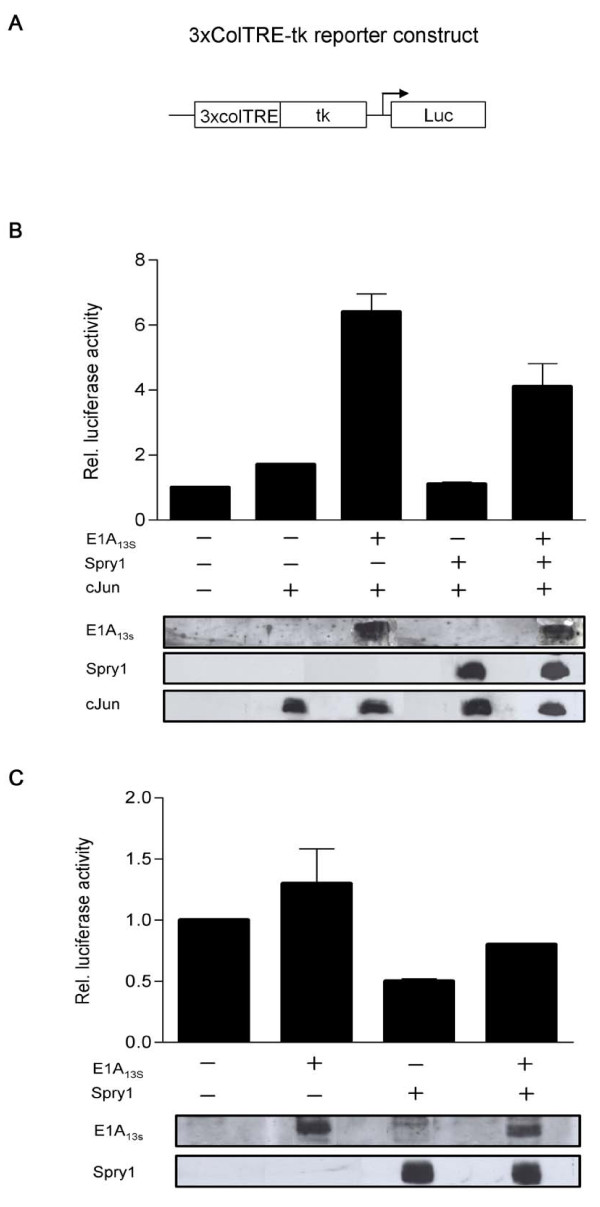
**E1A_13 _inhibits Spry1 down-regulation of the Col-TRE driven reporter gene**. (A) Schematic representation of the reporter construct 3xcolTRE-tk-Luc; the Luciferase expression is driven by the thymidine kinase promoter of the herpes simplex virus (tk, nt -105 to +51) and three copies of the TRE sequence of the human collagenase gene (colTRE, nt -73 to -65). (B, C) HeLa cells were co-transfected with the colTRE-Luc reporter construct (0.5 μg), expression vectors (0.5 μg) coding for E1A_13S _and/or Spry1 and c-Jun as indicated. Empty expression vectors were added to keep the amount of transfected DNA constant. 63 h after transfection cells were lysed and Luciferase activity was determined. Total lysates were immunoblotted to monitor the presence of expressed proteins with respective antibodies against E1A_13S_, Spry1 and c-Jun. Results are mean ± S.E.M. between duplicates.

**Figure 5:**
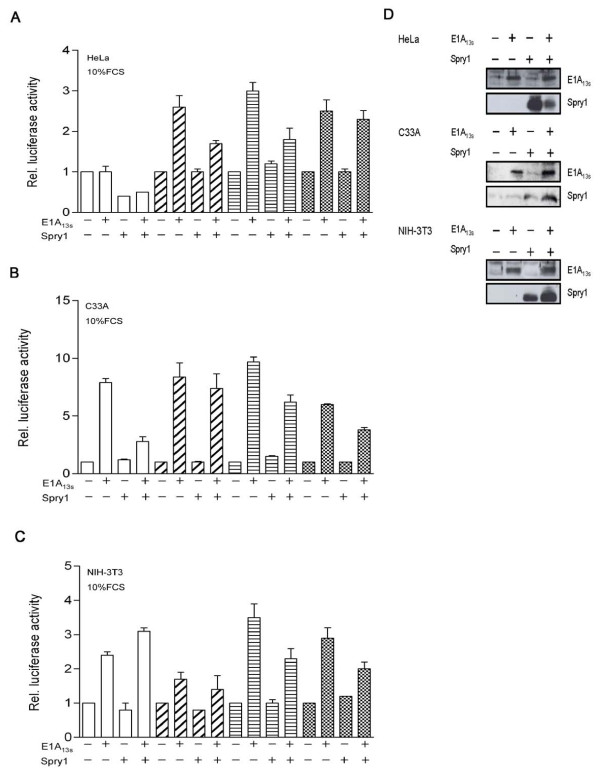
**Spry1 decreases E1A_13s _induced SRE transactivation in different cell lines**. (A) HeLa cells, (B) C33A cells and (C) NIH-3T3 cells were co-transfected with the SRE-Luc reporter construct (1 μg) and with plasmids (0.5 μg) expressing E1A_13S _and/or Spry1 as indicated. Empty expression vectors were added to keep the amount of transfected DNA constant. After transfection cells were left serum-deprived for 24 h and then incubated with 10% FCS in DMEM. Serum stimulation was performed for the indicated times (white, 1 h; cross line, 5 h; horizontal line, 7 h; dots, 9 h). The promotor activity of the reporter gene in the presence of empty vectors was set as 1. (D) Expression of Spry1 and E1A_13S _in the lysates was confirmed in Western blot assays. Results are mean ± S.E.M. between duplicates and representative for 3 individual experiments.

**Figure 6:**
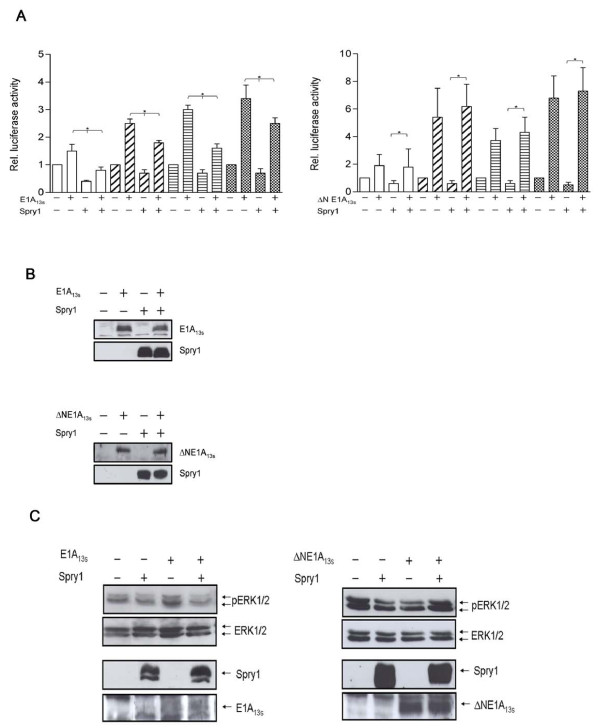
**Spry1 decreases wild type E1A_13S _induced gene expression but not ΔNE1A_13S _transactivation through ERK1/2 kinase phosphorylation**. (A) HeLa cells were co-transfected with the reporter construct SRE-Luc (1 μg) and plasmids (0.5 μg) expressing Spry1 and/or E1A_13S _or ΔNE1A_13S _as indicated. Empty expression vectors were added to keep the amount of the transfected DNA constant. After transfection cells were left serum-deprived for 24 h and then incubated with 20 ng/ml bFGF in DMEM. Serum stimulation was performed for the indicated times (white, 1 h; cross line, 5 h; horizontal line, 7 h; dots, 9 h). The promotor activity of the reporter gene in the presence of empty vectors was set as 1. Data represents mean ± S.E.M. of n ≥ 4. * p < 0.05 (B) Expression of Spry1 and E1A_13S _in the lysates was confirmed in Western blot assays. (C) HeLa cells expressing Spry1 and/or E1A_13S _or ΔNE1A_13S _were serum starved overnight, followed by incubation for 1 h with 20 ng/ml bFGF in DMEM. Cells were lysed and lysates were subjected to SDS-PAGE and analyzed by immunoblotting. The membranes were incubated with antibodies directed against phosphorylated ERK1/2 (phospho-ERK1/2), unphosphorylated ERK1/2 (ERK1/2) and antibodies directed against Spry1 and E1A_13S_.

**Figure 7:**
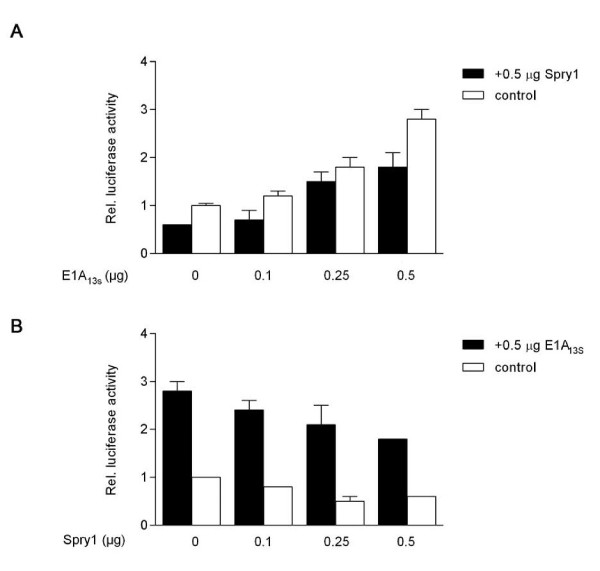
**Concentration-dependent suppression of Spry1 function by E1A_13S_**. HeLa cells were co-transfected with the SRE-Luciferase reporter construct and different concentrations of expression plasmids coding for Spry1 and/or E1A_13S_. We used either constant amounts of Spry1- (A) or E1A_13S_-expression vectors (B) in our experiments as indicated. Empty expression vectors were added to keep the amount of transfected DNA constant. After transfection cells were left serum-deprived for 24 h and followed by incubation with 20 ng/ml bFGF in DMEM. The promotor activity of the reporter gene in the presence of empty vectors was set as 1.

In transient expression assays we examined the effect of Spry1 and E1A_13S _interaction on gene expression activity. First, we analyzed this effect on the TPA-responsive element (TRE) in HeLa cells, which is transactivated by E1A and c-Jun [43,44]. Our results showed that the expression of the collagenase (Col)-TRE driven reporter gene is down-regulated after Spry1 expression and up-regulated due to the ectopic expression of E1A_13S_, whereas it is highly up-regulated when E1A_13S _and c-Jun are co-expressed. The E1A_13S_-induced gene transactivation was again repressed by co-expression with Spry1 (Figure 4). These data indicate that Spry1 decreases E1A_13S_-induced gene expression. Because Spry1 is known to act as a repressing factor in RTK signalling pathways, it is conceivable that co-expression of Spry1 represses E1A-induced gene expression by inhibiting the activity of specific kinases involved in transcriptional activation. To gain further insight into how the direct interaction of E1A_13S _with Spry1 functionally influences gene transactivation we decided to choose another reporter construct. The serum response element (SRE) is known to be downregulated by Spry proteins in response to bFGF treatment [23,45]. In our experiments cells were serum deprived for 24 h prior to stimulation with 10% FCS or 20 ng/ml bFGF for 1, 5, 7 and 9 hours. In HeLa cells, we detected a 2 to 3 fold increase in reporter gene activation in response to E1A_13S _expression after 5 - 9 h incubation with FCS, whereas in C33A and NIH-3T3 cell lines an upregulation of promoter activity by E1A_13S _was already detectable after 1 h of FCS stimulation (Figure 5). Similar results were obtained using bFGF instead of FCS for induction of the RTK signalling pathway after serum deprivation (Figure 6A). In cells that express both proteins, E1A_13S _abolishes the repression function of Spry1, whereby the E1A_13S_-induced gene expression activity consequently decreases by up to more than 50%. Concentration-dependent reporter assays showed a decrease of E1A_13S _promoter activation in response to an increase of Spry1 co-expression. Whereas the ability of Spry1 to reduce gene expression activity is abolished in response to an increase of E1A_13S _co-expression (Figure 7).

In co-expression experiments in which we expressed E1A_12S _instead of E1A_13S _however, we could not detect an increase in SRE-promoter activity and therefore no significant change after co-expression of Spry1 as well (data not shown).

Using the E1A_13S _N-terminal deletion mutant (ΔNE1A_13S_) in such transient expression assays, we were unable to detect a decrease of ΔNE1A_13S _induced gene expression in response to Spry1 compared with studies using wild type E1A_13S _(Figure 6). Also, the co-expression of ΔNE1A_13S _with Spry1 after 7 h and 9 h of stimulation showed a slightly higher SRE-dependent gene expression compared with cells expressing ΔNE1A_13S _exclusively. Our GST pull-down data showed that Spry1 interacts only weakly with ΔNE1A_13_, suggesting that the stronger interaction with Spry1 mediated by the N-terminus of E1A might be necessary for the inhibitory effect of Spry1 to repress E1A_13S _activity. Moreover it is worthwhile to note that these data also show that SRE can be activated by E1A_13S _independent of its N-terminal amino acids.

### Co-expression of Spry1 and E1A_13S _specifically impairs phosphorylation of ERK1/2 MAP kinase

It is reported that Spry1 inhibits the Ras/Raf/MAP kinase pathway [23]. To examine if the SRE-dependent reporter gene expression is affected by the ERK1/2 MAP kinase pathway, we analyzed the phosphorylation of ERK1/2 in HeLa cells after expression of Spry1 and E1A_13S _or ΔNE1A_13S_. A decrease in phosphorylation of ERK1/2 was detected in cells expressing Spry1, as compared with cells that were transfected with an empty expression plasmid, when visualized by phospho-specific antibodies. Expression of E1A_13S _in these cells led to an increased phosphorylation of ERK1/2 after 1 h of bFGF treatment, whereas the addition of Spry1 inhibited ERK1/2 phosphorylation (Figure 6C). For comparison we assayed the influence of ΔNE1A_13S _on ERK1/2 phosphorylation in the presence of Spry1. As expected, we were not able to detect an inhibition of the ERK1/2 phosphorylation/activation after co-expression of Spry1 and ΔNE1A_13S _(Figure 6C). Confirming the SRE reporter assays, a slight increase in phosphorylation was detectable in cells expressing Spry1 and ΔNE1A_13S _compared to cells only expressing ΔNE1A_13S_. To summerize, these results support our hypothesis of a functional interaction between E1A oncoproteins and Spry1 in the cytoplasm to modulate the Ras/Raf/MAP kinase pathway.

## Discussion

E1A oncoproteins have a key role in adenoviral replication. Their specific interaction with cellular proteins induces viral and cellular gene expression which initiates the host cell to enter S-Phase and therefore enables the virus life cycle to continue [46]. E1A binding partners are therefore specific targets that enable the virus to modulate the cell cycle. In this study, we identified Spry proteins as cytoplasmic interacting partners of adenoviral E1A proteins. Since Spry proteins are known as the "regulator" of RTK signalling pathway we studied and demonstrated the ability of E1A to modulate RTK signalling pathway through specific interaction with Spry1.

The mammalian Spry family consists of four Spry proteins. In our GST pull-down assays we showed differences in binding affinity of E1A isoforms with specific Spry family members (Spry1, Spry2, Spry4) which might reflect various interaction mechanisms and potential differences in functions of Spry isoforms. Here we observed a strong binding of Ad12 E1A proteins with Spry1 and Spry2, mediated via the aminoterminal E1A-sequence and furthermore the responsibility of CR3 for a less strong interaction with Spry1. Interestingly, the less conserved N-terminal sequence of the Ad12 E1A proteins is responsible for the interaction with two further cytoplasmic proteins, the 26S proteasom and the PKA-RIIα [14,17]. However, the amino acids involved in the interaction of these proteins are still unknown. Moreover we discovered the C-terminal half of Spry1, including the Spry domain, as the responsible region for E1A interaction. However, the Spry-domain-containing Spred proteins showed no interaction with E1A. It has yet to be clarified whether Spry-domain-neighbouring amino acids or variable amino acids within the conserved Spry domain mediate the interaction with E1A. Conformational changes between Spry and Spred proteins due to their Ena/Vasp homolog (EVH)1 and c-Kit binding (KBD)-domain, which is missing in Spry, may prevent E1A from binding [20].

We detected Spry1 accumulated and associated with E1A_13S _in vesicular structures within the whole cytoplasm. A localization of Spry proteins in vesicular structures has been reported before [22,47]. Palmitoylation targets Spry to the plasma membrane, which has been shown to be a necessary step for the inhibitory function of Spry in RTK signalling pathway [22,29].

Using TRE or SRE luciferase reporter assays we analyzed the functional consequences on gene expression activity by E1A_13S _and Spry1. The decrease of reporter gene expression by Spry1 was abolished when co-expressed with E1A_13S_. Expression of constant amounts of Spry1 and increasing amounts of E1A_13S _proteins showed that Spry1 proteins are functionally inactivated by E1A_13S _and vice versa. A functional repression of Spry1 would lead to an increasing activity of the RTK signalling pathway and to an increasing amount of phosphorylated transcription factors which could therefore enhance E1A-induced gene expression. This observation was supported by our experiments analyzing the phosphorylation of ERK. Overexpression of Spry1 decreased E1A_13S_-induced ERK-phosphorylation in comparison to the expression of E1A alone.

The up- or down-regulation of Spry has been described in different cancers [48], indicating the necessity of a balanced function of Spry proteins. Our data indicate that Spry1 is an important target of E1A proteins in the cytoplasm to modulate the RTK signalling pathway to influence cellular processes for optimizing viral replication.

Using the aminoterminal deletion mutant of E1A_13S _we obtained unexpected data. ΔNE1A_13S _displays only a weak binding with Spry1 via the CR3 in GST pull-down assays (Figure 1B) and can still increase reporter gene expression, whereas in co-expression with Spry1 no significant repression was detectable (Figure 6B). Possibly, the interaction via the CR3 has different effects on Spry1 function compared to the combined interaction via the N-terminus and the CR3 domain of E1A_13S_. These results were also supported by our phosphorylation studies of ERK, indicating an important N-terminal-dependent function of Ad12 E1A proteins for the interaction with Spry1. It is conceivable that the interaction mediated exclusively via the CR3 has a different effect on Spry1 function than the interaction mediated via the N-terminus and the CR3 domain of E1A_13S_. Instead of acting exclusively as inhibitors in signal transduction Spry proteins can be also involved in sustaining signal activity. This function dependents on the activity of binding partners such as c-Cbl [31,49,50]. The transcriptional gene expression activity of ΔNE1A_13S _and the interaction of Spry1 exclusively with the CR3 of E1A_13S _might cause a different influence on Spry1 function and would therefore explain our results using the E1A deletion mutant. Further studies are necessary to understand the mechanism of interaction between E1A and Sprouty proteins in detail.

## Conclusion

In conclusion, our results show for the first time that Spry proteins are targets of adenoviral E1A oncoproteins, which enables the virus to modulate the RTK signalling, leading to the ERK pathway, and to control, in addition to its transcriptional functions, cellular processes like proliferation, differentiation and apoptosis. The fact that Spry1 interacts with E7 of HPV 16 leads to the speculation that this might be a more general way of DNA viruses to modulate RTK signalling pathways. Over the past few years increasing evidence is implicating Spry in tumorgenesis and cancer [34,35,38]. Our identification and analysis of the functional interaction between the viral oncoprotein E1A and Spry support the idea of Spry being an important factor in tumorgenesis.

## Methods

### Cells, Growth Factors, Transfection Methods

HeLa, C33A and NIH-3T3 cells were cultured in Dulbecco's modified Eagle's medium with 10% fetal calf serum (FCS). For GST pull-down assay mouse Spry1, Spry2 and Spry4 were transfected by electroporation with the ECM830 electroporator. Cells were transfected by TransFectin Lipid reagent (Biorad) for co-immunoprecipitation and luciferase assays. For growth factor stimulation, cells were washed and maintained in serum-reduced medium (Dulbecco's modified Eagle's medium with 0.5% newborn calf serum) for 24 h prior to fetal calf serum/ bFGF (Invitrogen) treatment. Cells were harvested after several hours as indicated.

### DNA Constructs

The nucleotide sequence of murine Spry1, Spry2 and Spry4 are listed under AF176903, AF176905 and AF176906, respectively. Myc-tagged mouse Spry1 and Spry2 cDNAs cloned in plasmid pSG9M were a gift of Prof. G. Christofori (Basel, Swizerland) and the Myc-tagged mouse Spry4 cDNA cloned in plasmid pcDNA3 was a gift of Prof. A. Yoshimura (Fukuoka, Japan). Nucleotide sequences of GST-E1A-fusion proteins were cloned into the vector pGEX-2t (Pharmacia Biotech). Vector GST-E7pGEX-2t expressing the E7 protein of HPV16 was a gift of Prof. D.J. McCance (Rochester, New York). The N-terminal (residues 1-173) and C-terminal (residues 173-313) DNA fragments of mouse Spry1 and the Adenovirus E1A deletion mutants ΔNE1A_13S_, (residues 30-266) and ΔNE1A_12S_, (residues 30-235) were generated using standard polymerase chain reaction and molecular cloning methods. For co-immunoprecipitation, Spry1 was cloned via *BamHI-XhoI *sequences into the vector pcR3.1 (Invitrogen). E1A_13S _and E1A_12S _were cloned via *BamHI *sites into the vector pcDNA3.1/myc-His(-)B (Invitrogen). Mouse c-Jun was purchased from rzpd (clone ID: IRAVp968D0544D) and was cloned into the vector pcR3.1 (Invitrogen). The pGL3/Col-TRE reporter construct contains three colTRE-elements followed by the tk promoter of the herpes simplex virus, and the pGL3/SRE-Luc reporter construct contains five serum response elements (Stratagene). All constructs were sequenced.

### Immunoblot Analysis

Cells were lysed (10 mM Tris, pH 7.4, 5 mM MgCl_2_, 150 mM NaCl, 0.5% NP40, 1 mM 4-(2-Aminoethy1)-Benzene-SulfonylfluorideCl (Pefabloc SC) (Biomol)), proteins were separated by SDS-PAGE and transferred to Hybond-C Extra nitrocellulose membrane (Amersham Bioscience). The following primary antibodies were used: mouse monoclonal anti-Myc (Invitrogen); rabbit polyclonal anti-Sprouty1 (H120; Santa Cruz); rabbit polyclonal anti-c-JUN (H-79; Santa Cruz); rabbit anti-Ad12E1A antiserum (Genovac); rabbit polyclonal anti-phospho-p44/42 MAP kinase (#9101; NEB); rabbit polyclonal anti-p44/42 MAP kinase (#9102; NEB). For detection, one of the following second antisera were used: peroxidase goat anti-mouse IgG (H+L) (Pierce) at 1/50000; peroxidase goat anti-rabbit IgG (H+L) (Pierce) at 1/50000. Membranes were developed using ECL (Pierce; Amersham Pharmacia Biotech).

### GST pull-down assay

Glutathione Sepharose 4B was purchased from Amersham Bioscience. For preclearing, 0.5 mg of cellular lysate were incubated with 50 μl of Glutathion Sepharose (50%) for 1 h at 4°C. Subsequently, after washing by centrifugation at 500 × g for 5 min, supernatants were incubated with 40 μg of GST-fusion protein for 1 h at 4°C. The proteins bound were subjected to SDS-PAGE and immunoblot analysis was performed as described above.

### Co-Immunoprecipitation

Cells were lysed with RIPA buffer (Santa Cruz) and precleared with control IgG (Santa Cruz) and 20 μl of Protein A/G Plus-agarose (Santa Cruz). 0.5 mg of the cell lysates were incubated with 1.6 μg of the precipitating antibody for 1.5 h at 4°C while gentle rocking. 20 μl of Protein A/G Plus-agarose were added for overnight incubation. The beads were collected by centrifugation, washed 3 times with 1 ml of lysis buffer, and boiled in 40 μl 2 × SDS sample buffer. The immunoprecipitates were fractioned by SDS-PAGE and analyzed by immunobloting as described above.

### Immunofluorescence

HeLa cells (0.25 × 10^5^) were seeded onto sterilized glass coverslips contained in 24-well plates. After transfection, cells were maintained in serum-reduced medium overnight and stimulated with 20 ng/ml bFGF for various times. The cells were rinsed with PBS, fixed with 3% paraformaldehyde in PBS for 15 min at room temperature, permeabilized with 0.1% Triton X-100 for 4 min at room temperature, and washed with PBS. After blocking with 1% BSA/PBS for 30 min, cells were incubated with the primary antibody (mouse monoclonal anti-Myc (Invitrogen); rabbit polyclonal anti-Sprouty 1 (H120) (Santa Cruz)) for 1 h at room temperature. After washing with PBS, cells were incubated with the secondary antibody (Alexa Fluor 488 goat anti-rabbit IgG (Invitrogen); Cy3-conjugated goat anti-mouse IgG (dianova)) for l h at room temperature. After the final wash each coverslip was prepared for microscopic examination by applying mounting medium (Mowiol, Hoechst AG).

### Luciferase Assay

Cells were transfected by TransFectin Lipid reagent (Biorad) and Luciferase activity in cell lysates was measured by using the Promega-Luciferase assay system in a Berthold Lumat LB 9501 luminometer. In all reporter assays, 2.5 × 10^5 ^HeLa or C33A cells or 1.8 × 10^5 ^NIH-3T3 cells were plated on 6-well dishes.

### Statistics

All measured values are expressed as the mean ± S.E.M. The significance of the results was analyzed using Student's t-test.

## Competing interests

The authors declare that they have no competing interests.

## Authors' contributions

AZ carried out most of the experiments and wrote the manuscript. US participated in performing immunoblots and luciferase assays. HE was the supervisor of AZ, contributed to the intellectual design of the project and in the design of the manuscript. All of the authors read and approved the final version of this manuscript.
